# Synthesis of a new class of aminocyclitol analogues with the conduramine D-2 configuration

**DOI:** 10.3762/bjoc.6.15

**Published:** 2010-02-15

**Authors:** Latif Kelebekli, Yunus Kara, Murat Celik

**Affiliations:** 1 Atatürk University, Faculty of Science, Department of Chemistry, TR-25240, Erzurum, Turkey

**Keywords:** aminocyclitol, cyclitols, endoperoxide, oxidation, X-ray analysis

## Abstract

A new class of aminocyclitol derivatives with the bicyclo[4.2.0]octane skeleton was synthesized starting from cyclooctatetraene. Photooxygenation of *trans*-7,8-diacetoxy- and *cis*-7,8-dichlorobicyclo[4.2.0]octa-2,4-diene afforded the bicyclic endoperoxides. Reduction of the latter with thiourea followed by a Pd(0) catalyzed ionization/cyclization reaction gave the corresponding oxazolidinone derivatives. Oxidation of the double bond with KMnO_4_ or OsO_4_ followed by acetylation gave the acetate derivatives, the exact configuration of which was determined by spectroscopic methods. Hydrolysis of the oxazolidinone rings and removal of the acetate groups furnished the desired aminocyclitols.

## Introduction

Among the myriad of naturally occurring compounds are the aminocyclitol-containing natural products, which represent a large family of sugar derived microbial secondary metabolites and include the clinically active aminoglycoside inhibitors [1–11], many of which are widely used for the treatment of diseases in humans, animals and plants [1–15].

Glycosidase and related enzymes are involved in the biosynthesis of the oligosaccharide chains [1–15]. Carba analogues of oligosaccharides (carbasugars), generated by replacing the endocyclic O-atom in a monosaccharide [1–11], are thought to be better drug candidates than natural sugars, since they are hydrolytically stable. Spurred on by the heightened interest in the design of carbohydrate mimetics, which can be potent inhibitors of glycosidase (**1**–**4**) [11–16], we have developed a method for rapid entry to these compounds.


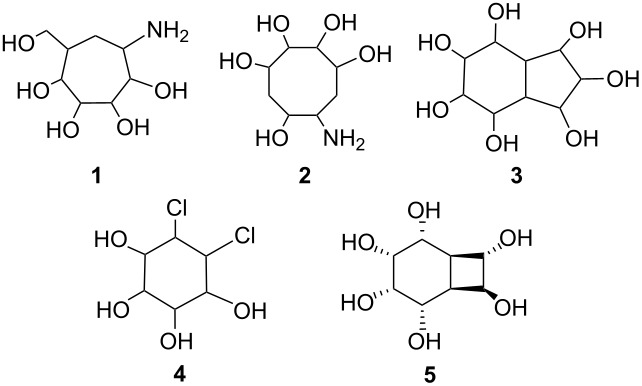


Antibiotics containing an aminocyclitol unit have stimulated the development of synthetic methodologies [16] in the search for analogues with enhanced pharmacological profiles [6]. Balci and Kara [20–22] have synthesized the polyhydroxylated bicyclic molecule **5** having the bicyclo[4.2.0]octane skeleton bis-homoinositol. Furthermore, Trost et al. [23–24] have reported a regio- and stereoselective Pd(0) catalyzed reaction of diols in the presence of *p*-toluenesulfonyl isocyanate for the introduction of the amino alcohol functionality.

We are currently interested in the synthesis of cyclitols and their derivatives [25]. As a part of our program directed towards the synthesis of potential glycosidase inhibitors we used a bicyclo[4.2.0]octane framework for OH, chlorine and NH_2_ groups as an intriguing carbohydrate alternative [26–30].

Herein, we report the synthesis of the new aminocyclitol analogues **6** and **7** from cyclooctatetraene.


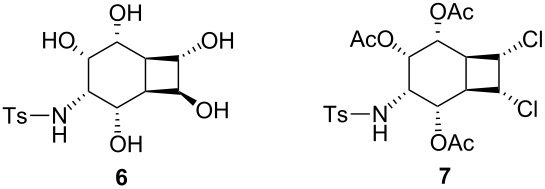


## Results and Discussion

**Scheme 1:**
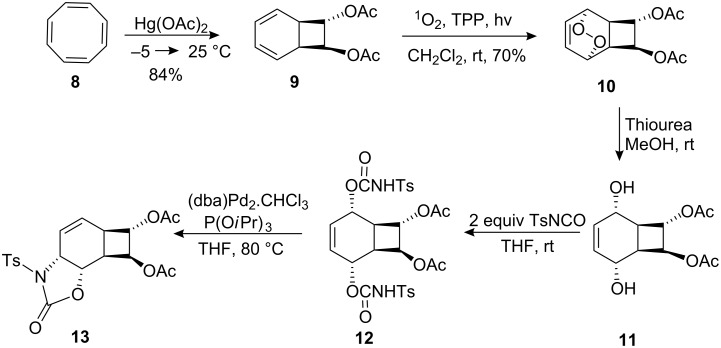
Synthesis of bis-carbamate **12** and oxazolidinone **13**.

Diacetoxydiene **9** was synthesized in 99% yield from cyclooctatetraene (**8**) by the addition of mercury(II) acetate [31]. Tetraphenylporphyrin sensitized photooxygenation of diacetoxydiene **9** with singlet oxygen gave the expected endoperoxide **10**. Reduction of the peroxide bond in **10** was performed with thiourea under very mild conditions to give the *cis*-diol **11** in 99% yield. The introduction of the amino alcohol functionality was achieved by a regio- and stereoselective Pd(0) catalyzed reaction of diol **11** and TsNCO [32]. Thus treatment of the *cis*-diol **11** in THF with 2 equiv of *p*-toluenesulfonyl isocyanate gave the corresponding bis-carbamate **12** which was subsequently added to a solution of 5 mol % of the catalyst, prepared by stirring a mixture of ligand (triisopropylphosphite) and tris(dibenzylideneacetone)–dipalladium-chloroform complex in THF. Subsequent purification by column chromatography gave oxazolidinone **13** in 48% yield (Scheme 1). The structure of **13** was assigned by ^1^H and ^13^C NMR and later by X-ray analysis of product **15**.

The observed regio- and stereoselectivity was remarkable since the leaving groups are diastereotopic.

**Scheme 2:**
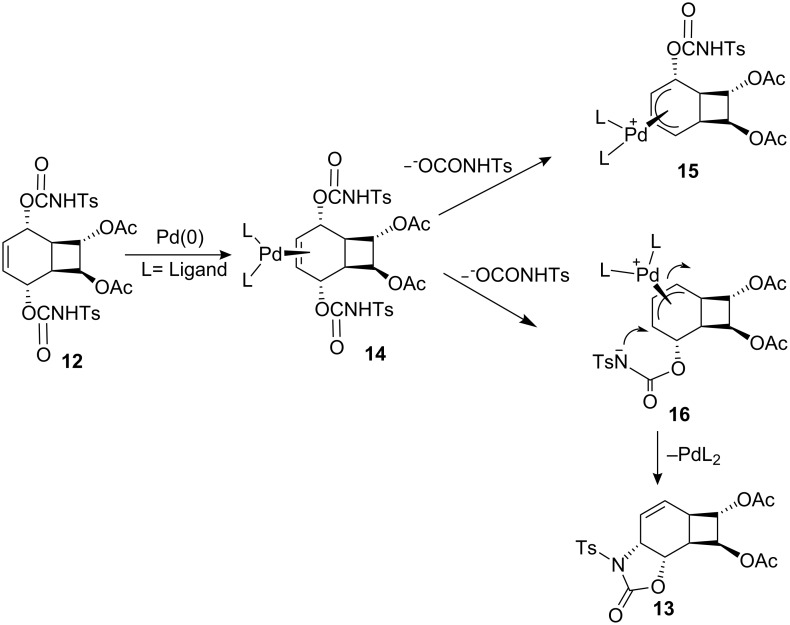
Mechanism of the palladium-catalyzed ionization/cyclization reaction.

The metal–olefin complexation is a likely source of the stereoselectivity. Mechanistically, only palladium–olefin complexation *anti* to the leaving group will lead to the product **13** [33–34], which is inconsistent with a steric preference for the metal approaching the double bond in **12** from the side of the four-membered ring to form complex **14** (Scheme 2).

Since the double bond is not symmetrically disubstituted, palladium can theoretically form two complexes **15** and **16** after ionization. We assume that the formation of complex **15** is hindered due to the presence of an acetate group in the *endo* position.

**Scheme 3:**
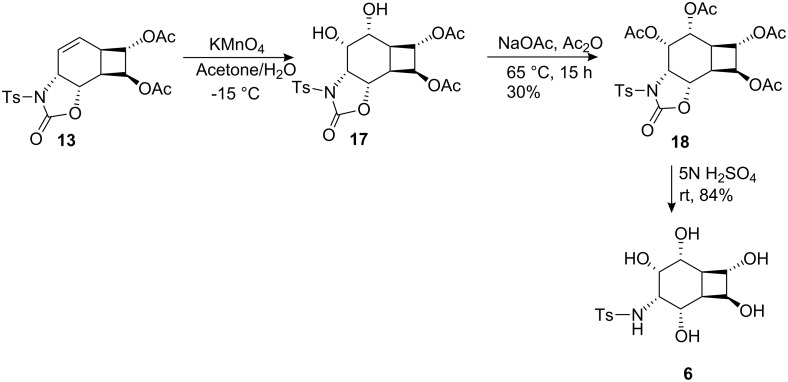
Synthesis of aminocyclitol analogue **6**.

**Figure 1:**
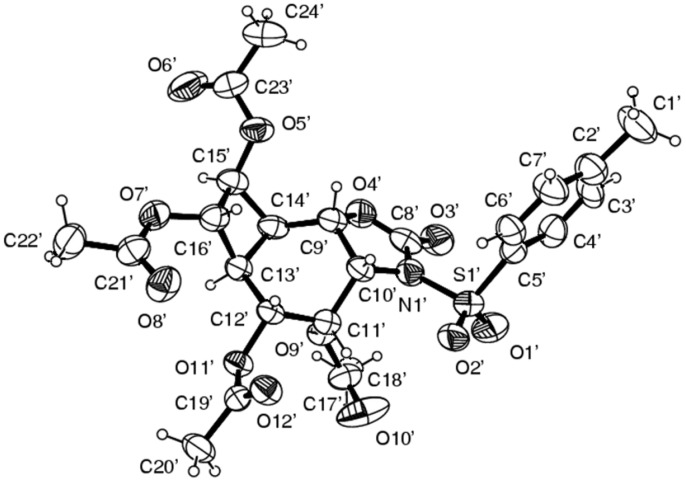
The thermal ellipsoid plot of the single crystal X-ray crystallographic structure of **18**.

*cis*-Dihydroxylation of **13** with KMnO_4_ at −15 °C gave a single diol **17**, which was converted into the tetraacetate by treatment with acetic anhydride/CH_3_COONa [35] (Scheme 3). Careful examination of the reaction mixture did not reveal the formation of the other isomer. The stereochemical course of the hydroxylation may be *syn* or *anti* with respect to the oxazolidinone and cyclobutane rings. NMR spectroscopic studies did not allow the assignment of the exact orientation of the hydroxyl groups. X-ray analysis of **18** (Figure 1) revealed the exact configuration of the compound. This also confirms the configurations of endoperoxide **10**, oxazolidinone **13** and *cis*-hydroxylation product **17**.

The all *cis*-configuration of the four acetate and amino groups in aminocyclitol **18** [25] attached to the six-membered ring resembles the configuration of conduramine D-2 [36–38]. Hydrolysis of the acetate groups with H_2_SO_4_ proceeded smoothly to deliver aminocyclitol **6** in 84% yield.

For the synthesis of dichloro derivative, we replaced the acetoxy groups in **9** with *cis*- configured chlorine atoms. This provides a route for the synthesis of other haloaminocyclitol derivatives.

**Scheme 4:**
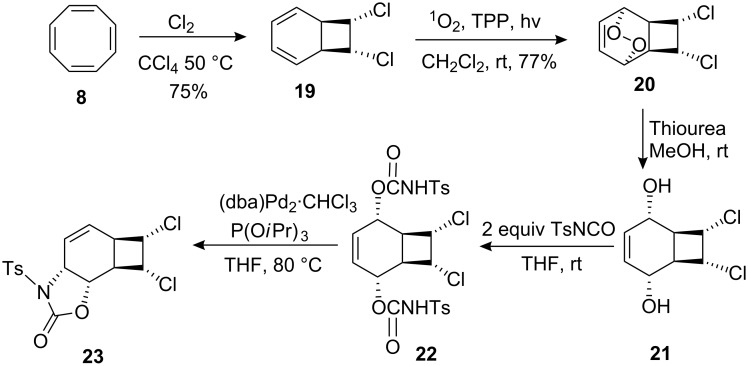
Synthesis of oxazolidone **23**.

*cis*-Dichlorobicyclooctadiene **19** was synthesized from cyclooctatetraene **8** by the addition of chlorine following the literature procedure [39]. Photooxygenation of *cis*-dichlorobicylooctadiene **19** with singlet oxygen gave the expected endoperoxide **20** [19–21] (Scheme 4). Since the dichlorobicyclooctadiene **19** has no plane of symmetry, singlet oxygen approaches the diene unit exclusively from the less crowded side of the molecule in accord with previous reports [20–21]. Reduction of the peroxide bond in **20** with thiourea under very mild conditions gave the *cis*-diol **21** in 95% yield. Diol **21** in THF was treated with 2 equiv of *p*-toluenesulfonyl isocyanate to give the intermediate bis-carbamate **22** which was then treated as described above for **12** with the same Pd(0) catalyst to afford, after chromatography on a silica gel with hexane/ethyl acetate (3:1) as eluant, the oxazolidinone **23** in 61% yield.

**Scheme 5:**
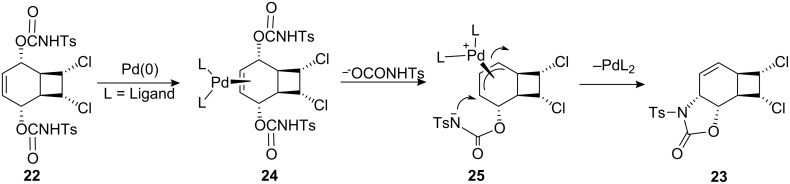
Mechanism of the palladium-catalyzed ionization/cyclization reaction in dichloro biscarbamate **22**.

The structure of **23** was assigned by ^1^H NMR and ^13^C NMR spectroscopy. The double bond in **22** is symmetrically disubstituted, and therefore palladium can form only one complex **25** after ionization (Scheme 5).

**Scheme 6:**
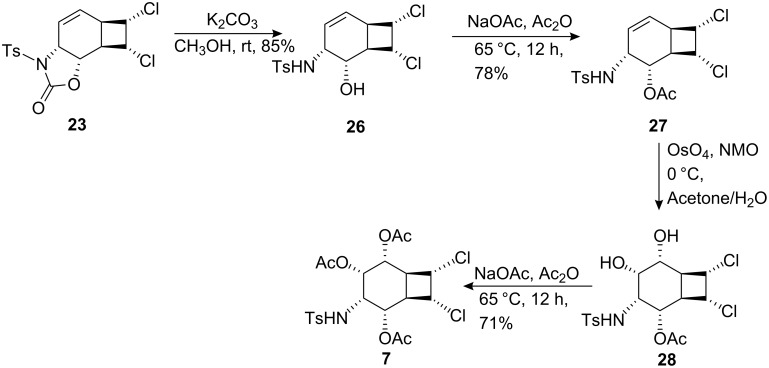
Synthesis of dichloroaminocyclitol **7**.

Hydrolysis of oxazolidinone **23** with K_2_CO_3_ gave alcohol **26**, which was subsequently converted into acetate **27** by treatment with Ac_2_O/NaOAc [35] (Scheme 6).

**Figure 2:**
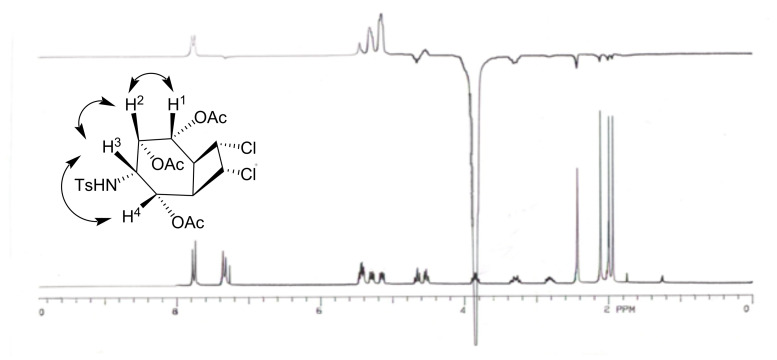
^1^H NMR NOE spectrum of compound **7**.

*cis*-Hydroxylation of **27** with OsO_4_ at 0°C gave the corresponding diol **28**, which was further converted into triacetate **7** with Ac_2_O/NaOAc (Scheme 6). The exact configuration of triacetate **7** was confirmed by differential ^1^H NMR NOE measurements (Figure 2). Irradiation of H^3^ (H^3^–CNHTs) at δ 3.85 caused signal enhancements of H^1^, H^2^, and H^4^ at δ = 5.45, 5.28 and 5.15, respectively. This result is consistent with a *cis*-relationship between H^1^, H^2^, H^3^ and H^4^ protons.

We assume that the stereochemical course of the hydroxylation of **27** proceeds through *syn* addition as previously observed in the hydroxylation of **13**, a quite similar structure to **27**.

The all *cis*-configuration of the acetate and amino groups attached to the six-membered ring resembles the configuration of conduramine D-2 [31,40–41]. The cyclic polyhdroxylated amines, also known as aminocyclitols, possess a wide variety of biological activities [42–45]. In conclusion, we have outlined the synthesis of a new family of aminocyclitols analogues **6** and **7** based on the bicyclo[4.2.0]octane frame work, with stereocontrol during the formation of all the stereogenic centres.

## Experimental

Melting points were determined on a Büchi 539 capillary melting apparatus and are uncorrected. Infrared spectra were obtained from KBr or film on a Mattson 1000 FT-IR spectrophotometer. The ^1^H and ^13^C NMR spectra were recorded on 200 (50) and 400 (100) MHz Varian spectrometer and are reported in δ units with SiMe_4_ as internal standard. Thin layer chromatography (TLC) was performed on E. Merck Silica Gel 60 F_254_ plate (0.2 mm). All column chromatography was performed on silica gel (60 mesh, Merck). Elemental analyses were carried out on a Carlo Erba 1108 model CHNS-O analyzer.

## Supporting Information

File 1 Experimental Section
The experimental section describes the synthesis, purification and characterization data of all substances given in this article.
