# Supplementary material for: Synthesis of a new class of aminocyclitol analogues with the conduramine D-2 configuration
Source: Beilstein J Org Chem. 2010 Feb 15;6:15. doi: 10.3762/bjoc.6.15 (PMC2870711; doi:10.3762/bjoc.6.15)
Supplement: File 1 — Experimental Section The experimental section describes the synthesis, purification and characterization data of all substances given in this article. [file Beilstein_J_Org_Chem-06-15-s001.pdf]

Supporting Information

for

Synthesis of a new class of aminocyclitol analogues with the conduramine D-2 configuration

Latif Kelebekli, Yunus Kara* and Murat Celik*

Address: Atatürk University, Faculty of Science, Department of Chemistry,
TR-25240, Erzurum, Turkey

Email: Murat Celik* - mcelik@atauni.edu.tr
Yunus Kara* - yukara@atauni.edu.tr

*Corresponding author

Experimental

Synthesis of *rac-(1R,6S,7S,8S)-8-(acetyloxy)bicyclo[4.2.0]octa-2,4-dien-7-yl acetate* (9).

The title compound was prepared in 84% yield as described in the literature [1,2].

Synthesis of *rac-(1R,2S,3S,4S,5R,6S)-4-(acetyloxy)-7,8-dioxatricyclo[4.2.2.0^{2,5}] dec-9-en-3-yl acetate* (10).

The title compound was prepared in 70% yield as described in the literature [3].

Synthesis of *rac-(1R,2R,5S,6S,7S,8S)-8-(acetyloxy)-2,5-dihydroxy-bicyclo[4.2.0] oct-3-en-7-yl acetate* (11).

To magnetically stirred slurry of 0.30 g (3.93 mmol) of thiourea in 20 mL of methanol was added a solution of 1.00 g (3.93 mmol) of diacetyloxy-endoperoxide **10** in 50 mL of mixture of CH_2Cl_2/CH_3OH (1 : 9) at room temperature. After the addition was

complete (ca. 10 min) the mixture was stirred for 1 h and the solid removed by filtration. Evaporation of the solvent gave diol **11** as a colorless oil (1.00 g, 99%); δ_H (200 MHz, CD_3OD) 5.89 (dt, A part of AB-system,1H, J_{AB} = 10.3, 2.0 Hz, C=CH), 5.80 (dt, B part of AB-system,1H, J_{AB} = 10.3, 2.0 Hz, C=CH), 5.17 (t, 1H, J = 7.3 Hz, –CH–OH), 4.85 (t, 1H, J = 7.3 Hz, –CH–OH), 4.24 (m, 2H, –CH–OAc), 2.54 (m, 1H, –CH), 2.30 (m, 1H, –CH), 2.08 (s, 3H, $–CH_3$), 2.07 (s, 3H, $–CH_3$); δ_C (50 MHz, CD_3OD) 174.1, 173.8, 136.4, 133.3, 78.8, 73.8, 67.4, 65.2, 43.9, 39.3, 22.7(×2).

Synthesis of *rac-(3aR,5aR,6S,7aS,7bS)-6-(acetyloxy)-3-(p-toluenesulfonamido)-2,3,3a,5a,6,7,7a,7b-octahydrocyclobuta[g][1,3]benzoxazol-7-yl acetate* (13).

To a stirred solution of diol **11** (1.0 g, 3.9 mmol) in anhydrous THF (20 mL) under a nitrogen atmosphere at room temperature, *p*-toluenesulfonyl isocyanate (1.55g, 1.18 mL, 7.81 mmol) was added dropwise. The reaction mixture was stirred at room temperature for 5 h and then at 80 °C for 60 min. To a flask containing tris(dibenzylidene-acetone)dipalladium–chloroform complex (0.25 g, 242 μmol) in anhydrous THF (10mL) under a nitrogen atmosphere was added triisopropylphosphite (0.40 g, 1.94 mmol). The mixture was stirred at room temperature for 30 min until a clear yellow solution was obtained. The reaction mixture was then stirred at 80 °C for 24 h. After removal of the solvent under reduced pressure (50 °C, 20 mmHg), the mixture was chromatographed on silica gel (60 g) with 35% ethyl acetate/hexane as eluant to afford **13** (0.82 g, 48%). White crystals, mp 144–146 °C (from hexane/ethyl acetate). Found: C, 55.36; H, 5.01; N, 3.09; S, 7.19; $C_{20}H_{21}NO_8S$ requires C, 55.16; H, 4.86; N, 3.22; S, 7.36; ν_{max} (KBr) 2978, 2953, 1804, 1753, 1395, 1268, 1242, 1165, 1063, 680, 604 cm^{-1}; ^1H NMR (200 MHz, $CDCl_3$) δ_H 7.94 (br d, A part of AA′BB′ system, J = 8.3 Hz, 2H, aromatic), 7.34 (d, B part of AA′BB′ system, J = 8.3 Hz, 2H, aromatic), 6.24 (ddd, A part of AB system,

$J_{4,5}$ = 10.3, $J_{5,5a}$ = 4.5, and $J_{5,3a}$ = 1.1 Hz, 1H, H-5), 6.01 (br d, B part of AB system, $J_{4,5}$ = 10.3 Hz, 1H, H-4), 5.24 (ddd, 1H, $J_{7b,3a}$ = 9.2, $J_{7b,7a}$ = 4.7, $J_{7b,5a}$ = 1.3 Hz, 1H, H-7b), 4.75–4.80 (m, 2H, H-3a and H-7), 4.42 (dt, $J_{6,5a}$ = $J_{6,7}$ = 5.6, and $J_{6,7a}$ = 1.1 Hz, 1H, H-6), 3.14 (br t, $J_{7a,7}$ = $J_{7a,5a}$ = 9.2 Hz, 1H, H-7a), 2.70 (br dt, $J_{5a,7a}$ = 9.2 Hz, and $J_{5a,5}$ = $J_{5a,6}$ = 5.5 Hz, 1H, H-5a), 2.45 (s, 3H, arom–CH_3), 2.09 (s, 3H, OC–CH_3), 2.07 (s, 3H, OC–CH_3); ^{13}C NMR (50 MHz, $CDCl_3$) 171.2 (s), 170.8 (s), 152.5 (s), 147.2 (s), 137.4 (s), 131.7 d (2×), 130.6 (d), 124.7 (d), 80.6 (d), 72.0 (d), 71.3 (d), 55.9 (d), 35.9 (d), 33.5 (d), 23.7 (q), 22.5 (q, 2×).

Synthesis of *rac-(3aS,4S,5R,5aR,6S,7R,7aR,7bS)-4,5,6-tri(acetyloxy)-3-(p-toluenesulfonamido)-decahydrocylobuta[g][1,3]benzoxazol-7-yl acetate* (18). To a stirred solution of 0.85 g (1.87 mmol) **13** in acetone (80 mL) was added a solution of $KMnO_4$ (0.31 g, 1.87 mmol) and $MgSO_4.H_2O$ (0.30 g, 1.87 mmol) in water (30 mL) at −15 °C over a period of 5 h. After the addition was complete, the reaction mixture was stirred for an additional 15 h at −15 °C and then filtered. The precipitate was washed several times with hot water, and combined filtrates were concentrated (60 °C, 20 mmHg) to 20 mL. The aqueous solution was extracted with ethyl acetate (3 × 75 mL) and the extracts dried over Na_2SO_4. Evaporation of the solvent gave crude **17**, which was dissolved in 20 mL of acetic anhydride and to this magnetically stirred solution was added excess of CH_3COONa. The reaction mixture was stirred for 15 h at 90 °C. The mixture was cooled to 0 °C and 100 mL of 1 M HCl solution was added. The mixture was extracted with ether (3 × 75 mL). The combined organic extracts were washed with sat. Na_2CO_3 solution (20 mL), water (50 mL) and then dried over Na_2SO_4. After the removal of the solvent under reduced pressure (30 °C, 20 mmHg), the mixture was separated by thin-layer chromatography (35% ethyl acetate/hexane) to afford **18** (0.315 g, 30%). White crystals, mp 105–107 °C (from

CH_3OH). Found: C, 51.68; H, 4.91; N, 2.69; S, 5.69; $C_{24}H_{27}NO_{12}S$ requires C, 52.08; H, 4.92; N, 2.53; S, 5.79; ν_{max} (KBr) 3004, 2927, 1753, 1651, 1525, 1523, 1446, 1395, 1242, 1191, 1114, 1089, 936 cm^{-1}; ^1H NMR (200 MHz, $CDCl_3$) δ 7.87 (d, A part of AA′BB′ system, J_{AB} = 8.3 Hz, 2H, aromatic), 7.27 (d, B part of AA′BB′ system, J_{AB} = 8.3 Hz, 2H, aromatic), 5.70 (dd, J = 3.4 and 1.8 Hz, 1H, O–CH), 5.20 (dd, J = 7.7 and 6.2 Hz, 1H), 5.05 (dd, J = 6.5 and 1.6 Hz, 1H), 4.94 (dd, J = 9.0 and 6.0 Hz, 1H), 4.83 (br t, J = 6.4 Hz, 1H), 4.75 (dd, J = 9.2 and 3.4 Hz, 1H), 3.13 (m, 1H, H_{5a} or H_{7a}), 2.39 (s, 3H, arom–CH_3), 2.30 (m, 1H, H_{5a} or H_{7a}), 2.04 (s, 3H, OC–CH_3) 2.00 (s, 3H, OC–CH_3), 1.97 (s, 3H, OC–CH_3) 1.79 (s, 3H, OC–CH_3); ^{13}C NMR (50 MHz, $CDCl_3$) 171.9 (s), 171.7 (s), 171.1 (s), 171.0 (s), 153.3 (s), 147.8 (s), 136.4 (s), 131.7 (d), 130.6 (d), 77.9 (d), 72.7 (d), 71.3 (d), 71.2 (d), 69.7 (d), 59.1 (d), 39.0 (d), 38.2 (d), 23.6 (q), 22.6 (q), 22.5 (q) (2C), 22.4 (q).

Synthesis of *rac-(1S,2R,3S,4S,5S,6R,7S,8S)-4-(p-toluenesulfonamido) bicyclo[4.2.0]octane-2,3,5,7,8-pentol: bis-homoamino-inositol analogue* (6).
18 (0.11 g, 0.199 mmol) was dissolved in 5 mL of 0.5 N H_2SO_4. The resulting mixture was stirred at room temperature for 5 h and the acid neutralized with $BaCO_3$. The solid material was filtered and the filtrate concentrated under reduced pressure to yield the *bis*-homoaminoinositol analogue **6** (60 mg, 84%). Colorless powder, mp 177–179 °C (from hexane/ethyl acetate); ν_{max} (KBr) 3401, 2931, 1735, 1450, 1234, 1157, 1103, 1049, 1010, 856, 817, 748, 671, 578, 555 cm^{-1}; Found: C, 49.98; H, 5.71; N, 3.79; S, 30.81. $C_{15}H_{21}NO_7S$ requires C, 50.13; H, 5.89; N, 3.90; S, 31.16; ^1H NMR (200 MHz, CD_3OD) δ 7.79 (d, A part of AA′BB′ system, J = 8.3 Hz, 2H, aromatic), 7.33 (d, B part of AA′BB′ system, J_{AB} = 8.3 Hz, 2H, aromatic), 4.91 (br s, 6H, –OH and –NH), 4.00–3.54 (m, 6H, O–CH and N–CH), 2.41 (s, 3H, arom–CH_3), 2.31 (m, 1H, C–CH), 2.04 (m, 1H, C–CH); ^{13}C NMR (50 MHz, CD_3OD) 146.3 (s),

141.5 (s), 132.2 (d), 130.3 (d), 77.8 (d), 75.3 (d), 71.7 (d), 70.9 (d), 70.2 (d), 62.8 (d), 45.0 (d), 38.5 (d) , 23.2 (q).

Synthesis of *1R,6S,7S,8R-7,8-dichlorobicyclo[4.2.0]octa-2,4-diene* (19).

The title compound was prepared in 75% yield as described in the literature [4,5].

Synthesis of *1R,2S,3S,4R,5R,6S-3,4-dichloro-7,8-dioxatricyclo [4.2.2.0^{2,5}]dec-9-ene* (20).

The title compound was prepared in 75% yield as described in the literature [6,7].

Synthesis of *-1R,2S,5S,6S,7S,8R-7,8-dichlorobicyclo[4.2.0]oct-3-ene-2,5-diol* (21).

The title compound was prepared in 95% yield as described in the literature [6,7].

Synthesis of *rac-(3aR,5aR,6S,7R,7aR,7bS)-6,7-dichloro-3-tosyl-3a,5a,6,7,7a,7b-hexahydrocyclobuta[g][1,3]benzoxazol-2(3H)-on* (23).

To a stirred solution of diol **21** (1.1 g, 5.28 mmol) in anhydrous THF (20 mL) under a nitrogen atmosphere at room temperature, p-toluensulfonyl isocyanate (2.80 g, 1.60 mL, 10.55 mmol) was added dropwise. The reaction was stirred at room temperature for 5 h and then at 80 °C for 60 min. To a flask containing tris(dibenzylidene-acetone)dipalladium chloroform complex (0.25 g, 242 μmol) in anhydrous THF (10 mL) under a nitrogen atmosphere was added triisopropylphosphite (0.40 g, 1.94 mmol). The mixture was stirred at room temperature for 30 min until a clear yellow solution was obtained. The reaction mixture was stirred at 80 °C for 24 h. After the removal of the solvent under reduced pressure (50 °C, 20 mmHg), the mixture was chromatographed on silica gel (60 g) with 35% ethyl acetate/hexane as eluant to afford oxazolidin-2-one-diacetate **23**

(1.25 g, 61%). White crystals, mp 180–183 °C (from $CHCl_3$/hexane); ν_{max} (KBr) 3029, 2979, 2933, 1782, 1597, 1493, 1454, 1366, 1327, 1170, 1135, 1096, 1023, 858, 842, 815, 758, 727, 673, 592 cm^{-1}. ^1H NMR (200 MHz $CDCl_3$ ppm) δ 7.96 (d, A part of AA ′BB′ system, J = 8.3 Hz, 2H, aromatic), 7.37 (d, B part of AA′BB′ system, J = 8.3 Hz, 2H, aromatic), 6.10 (bs, 2H, –CH=CH), 4.94 (dd, A part of AB system, J = 7.3, 1.8 Hz, 1H, H_{3a}, -CH-N), 4.84 (dd, B part of AB system, J = 7.3, 2.1 Hz, 1H, H_8, –CH–O), 4.32 (ddd, $J_{7,7a}$ = 9.6 Hz, $J_{6,7}$ = 6.3 Hz, J = 1.9 Hz, 1H, H_7, CH–Cl), 4.22 (bd, J = 6.3 Hz, 1H, H_6, CH–Cl), 3.52 (ddd-quasi td, J = 9.6, 9.0, 2.0 Hz, 1H, H_{7a}, –CH), 3.01 (bd, J = 9.0 Hz, 1H, H_{5a}, –CH), 2.45 (s, 3H, arom–CH_3).

^{13}C NMR (50 MHz $CDCl_3$ ppm) δ 152.3 (s), 147.8 (s), 137.1 (s), 131.9 (d), 130.5 (d), 130.4 (d), 125.2 (d), 72.1 (d), 62.5 (d), 54.8 (d), 54.4 (d), 43.5 (d), 39.5 (d), 23.7 (q). EIMS (m/z %): 389 (M^+, 2.5), 279.7 (11), 211.6 (7), 208.4 (9), 207.3 (44), 167.5 (25), 149.3 (100), 148.5 (33), 83.3 (10), 71.2 (24.5), 57.2 (17).

Synthesis of *rac-(1R,2S,3R,6R,7S,8R)-7,8-dichloro-3-(4-methylbenzenesulfonamido)-bicyclo[4.2.0]oct-4-en-2-yl acetate* (27).

To a stirred solution of oxazolidin-2-one **23** (0.75 g, 1.93 mmol) in methanol (15 mL) was added K_2CO_3 (0.80 g, 1.93 mmol) and the mixture stirred at room temperature for 6 h. The reaction mixture was filtered and the solvent removed to give crude product **26** (0.70 g). Crude **26** was dissolved in 15 mL of acetyl chloride and the resulting solution stirred at room temperature overnight. The excess of unreacted acetyl chloride was evaporated (60 °C, 20 mmHg). The residue was dissolved in CH_2Cl_2 and filtered through silica gel. Evaporation of the solvent gave **27** (0.71 g, 90%). White crystals, mp 180–182 °C (from $CHCl_3$/hexane); ν_{max} (KBr) 3270, 3031,

2962, 1739, 1600, 1438, 1334, 1238, 1160, 1091, 1041, 937, 813, 763, 678, 551 cm^{-1}.

^{1}H NMR (200 MHz $CDCl_3$ ppm) δ 7.75 (d, A part of AA′BB′ system, *J* = 8.3 Hz, 2H, aromatic), 7.32 (d, B part of AA′BB′ system, *J* = 8.3 Hz, 2H, aromatic), 5.89 (ddd, A part of AB system, *J* = 10.0, 3.8, 1.6 Hz, 1H, H_5, –CH=CH), 5.51 (ddd, B part of AB system, *J* = 10.0, 4.36, 1.6 Hz, 1H, H_4, –CH=CH), 5.37 (d, *J* = 9.1 Hz, 1H, N–H), 4.90 (dd, *J* = 7.6, 4.2 Hz, 1H, H_2), 4.28 (m, 2H, –CH–Cl), 4.05 (m, 1H, H_3, CH–N), 3.19 (m, 1H, H_6), 3.02 (m, 1H, H_1), 2.43 (s, 3H, arom–CH_3), 2.00 (s, 3H, OC–CH_3). ^{13}C NMR (50 MHz $CDCl_3$ ppm) δ 172.3 (s), 145.8 (s), 140.0 (s), 131.9 (d), 130.6 (d), 129.5 (d), 128.9 (d), 70.8 (d), 61.8 (d), 58.2 (d), 50.3 (d), 44.2 (d), 43.9 (d), 23.5 (q), 22.8 (q). EIMS (*m/z*, %): 401 (M^+, 1), 355 (31), 281 (28), 221 (35), 207 (40), 147 (59), 73 (100), 44 (17).

Synthesis of *rac-(2R,3S,4S,5S,7R,8S)-7,8-dichloro-4-(4-methylbenzenesulfonamido)bicyclo[4.2.0]octane-2,3,5-triyl triacetate* (7).

A 100 mL two-necked, round-bottomed flask, equipped with a magnetic stirrer and a nitrogen inlet, was charged with 0.28 g (2.37 mmol) of NMO, 1 mL of water, and 3 mL of acetone. To this solution were added ca. 2 mL of OsO_4 (250 g/60 mL solution in acetone) and 0.96 g (2.37 mmol) of *cis*-dichloro-acetate **27**. The resulting mixture was stirred vigorously under a nitrogen atmosphere at 0 °C and then stirred overnight. After stirring for 18 h, sodium bisulfite (100 mg) and 2 g of Florisil slurried in 1 mL of water were added, the slurry stirred for 1 h, and the mixture filtered through a short pad (2.0 g) of Celite in a 50 mL sintered glass funnel. The Celite cake was washed with acetone (3 × 10 mL). The filtrates were combined and solvent was removed to give crude *cis*-dichloro-acetate-diol **28**. The same procedure as

described above was applied for acetylation of **28**. After the removal of the solvent under reduced pressure (50 °C, 20 mmHg), the mixture was chromatographed on silica gel (60 g) with 40% ethyl acetate/hexane as eluant to afford triacetate **7** (0.31 g, 86%). White crystals, mp 155–157 °C (from $CHCl_3$/hexane); ν_{max} (KBr) 3276, 3025, 2921, 1747, 1597, 1443, 1374, 1335, 1227, 1162, 1096, 1046, 981, 942, 765 cm^{-1}. ^{1}H NMR (200 MHz CD_3OD ppm) δ 7.76 (d, A part of AA′BB′ system, *J* = 8.3 Hz, 2H, aromatic), 7.34 (d, B part of AA′BB′ system, *J* = 8.3 Hz, 2H, aromatic), 5.44 (dd, *J* = 7.1, 3.6 Hz, 1H, H_2), 5.29 (dd, *J* = 7.6, 3.3 Hz, 1H, H_3), 5.23 (d, *J* = 7.1 Hz, 1H, –NH–Ts), 5.15 (dd, *J* = 6.5, 4.0 Hz, 1H, H_5), 4.64 (t, 1H, *J* = 6.6 Hz, H_8, –CH–Cl), 4.53 (t, 1H, *J* = 5.6 Hz, H_7, –CH–Cl), 3.86 (ddd, *J* = 9.3, 7.3, 3.9 Hz, 1H, CH–N), 3.29 (m, 1H, H_1), 2.84 (m, 1H, H_6), 2.43 (s, 3H, arom–CH_3), 2.12, 1.99, 1.95 (s, 9H, OC–CH_3). ^{13}C NMR (50 MHz $CDCl_3$ ppm) δ 171.7 (x2, s), 171.6 (s), 146.1 (s), 138.9 (s), 131.9 (d), 129.1 (d), 71.4 (d), 70.6 (d), 66.8 (d), 59.9 (d), 57.5 (d), 53.7 (d), 46.2 (d), 44.0 (d), 23.5 (q), 22.9 (×3, q).

EIMS (*m/z*, %): 519 (M^+, 1), 429 (4), 355 (18), 281 (45), 221(30), 207 (93), 73 (100), 44 (55).

References

1. Trost, B. M.; Van Vranken, D. L.; Birgel, C. *J. Am. Chem. Soc.* **1992,** *114,* 9327. doi:10.1021/ja00050a013
2. Supplementary data in the form of CIFs have been deposited with the Cambridge Crystallographic Data Centre (CCDC 299509). Copies of the data can be obtained, free of charge, on application to CCDC, 12 Union Road, Cambridge CB2 IEZ, UK [Fax: +44(0)-1223-336033 or e-mail: deposit@ccdc.cam.ac.uk]. Selected X-ray crystallographic data for **19** ($C_{24}H_{27}NO_{12}S$): Space group: Orthorhombic, *Pbn*21; *a* = 11.6906(4) Å, b = 20.8899(10) Å, c = 22.1919(9) Å, *V* = 5419 Å^3, Z = 8,F(000) = 2320, D_{calc} =

1.36 g cm^{-3}, MoK$_{\alpha}$ = 0.71073 Å, independent reflections 7705 (Rint = 0.0421), λ radiation observed reflections 7093 (I > 2rI), refinement method; full-matrix least-squares on *F*2, data/restraints/parameters 7093/1/695, R_1 = 0.0557, R_w = 0.1186, goodness-of-fit on *F*2 = 1.21.

3. Kelebekli, L.; Celik, M.; Kara, Y.; Balci, M. *Tetrahedron Lett.* **2006,** *47,* 7031.doi:10.1016/j.tetlet.2006.07.108
4. Leung-Toung, R.; Liu, Y.; Muchowski, J. M.; Wu, Y.-L. *Tetrahedron Lett.* **1994,** *35,* 1639.doi:10.1016/0040-4039(94)88307-6
5. Leung-Toung, R. Y.; Liu, J.; Muchowski, J. M.; Wu, Y.-L. *J. Org. Chem.* **1998,** *63,* 3235.doi:10.1021/jo971907r
6. Kelebekli, L.; Kara, Y.; Balci, M. *Carbohydr. Res.* **2005,** *340,* 1940.doi:10.1016/j.carres.2005.05.021
7. Kara, Y.; Balci, M. *Tetrahedron* **2003,** *59,* 2063.doi:10.1016/S0040-4020(03)00209-6
